# Incidence trends and epidemiology of invasive device-associated bacteremia in French nursing home residents, 2020–2024: Insights from the SPIADI Prospective Multicenter Study

**DOI:** 10.1007/s10096-025-05188-4

**Published:** 2025-06-23

**Authors:** on behalf of the members of the SPIADI Network, Maris Dussartre, Nicolas Duflot, Anne-Sophie Valentin, Mathilde Farizon, Florent Goube, Nathalie van der Mee-Marquet

**Affiliations:** Mission Nationale Surveillance et Prévention des Infections Associées aux Dispositifs Invasifs (SPIADI)Appui Pour La Prévention Des Infections Associées Aux Soins en Région Centre Val de LoireHôpital Bretonneau, Centre Hospitalier Régional Universitaire, Hôptal Bretonneau, Tours, France

**Keywords:** Nursing home, Intravascular devices-related bacteremia, Urinary devices-related bacteremia, Central line associated bacteremia, Central venous catheter, Implantable port catheter, Peripherally inserted central catheter

## Abstract

Healthcare-associated bacteremia is associated with increased morbidity and mortality. In nursing homes, these infections remain under-documented. We investigated invasive device-associated bacteremia in residents. We analyzed bacteremias acquired in nursing homes using data from a national surveillance program conducted between 2020 and 2024, involving 1,233 French healthcare institutions. A total of 2,117 bacteremias acquired in the nursing home were recorded. The main sources of infection were the urinary tract (52.1%) and the respiratory tract (11.9%). An invasive device was involved in 20.0% of cases, primarily urinary catheters (386 cases), while bacteremia related to intravascular devices was rare (38 cases). *Enterobacterales* (64.0%) and *Staphylococcus aureus* (14.6%) were the most frequently identified pathogens, with multidrug-resistant bacteria detected in 15.2% of nursing home-acquired bacteremias. The incidence rate was 0.009 per 1,000 resident-days, remaining stable over the study period. The study highlights the burden of bacteremias in nursing homes and underscores the importance of targeted infection prevention measures, particularly in relation to urinary catheter management, and long-term intravascular central lines.

**Supplementary information:**

The online version contains supplementary material available at 10.1007/s10096-025-05188-4.

## Introduction

Healthcare-associated bacteremia is associated with increased morbidity, mortality, and healthcare costs, particularly among the most vulnerable individuals [1, 2]. Moreover, due to the growing issue of antibiotic-resistant bacteria, bacteremia is becoming increasingly difficult to treat.

The risk factors for these infections are well established and include both patient-related factors (e.g., extreme age, impaired immunity, underlying diseases such as advanced cancer, or prolonged hospitalization) and healthcare-related factors (e.g., use of invasive devices, recent antibiotic exposure, non-compliance with infection prevention measures, and respiratory support) [3–5]. The 2023–2024 HALT-4 point prevalence survey of infections, conducted in 1,662 European long-term care facilities, confirmed that bacteremia remains a relevant concern in nursing homes today, as resident-related risk factors are clearly present in current populations [6]. In the 44 French nursing homes that participated in this study, 3,140 residents were surveyed [7]. The median age was 78.5 years, with 64.1% over 85 years old. Women accounted for 71.7% of the population, 66.1% had urinary and/or fecal incontinence, 68.1% were cognitively impaired, and 45.4% had reduced mobility. On the day of the survey, one in 85 residents had a urinary catheter, and one in 45 residents had an intravascular device. HALT-4 reported that 2.4% of French residents had an active infection, with urinary tract infections accounting for 23.4%, respiratory tract infections for 31.2%, and skin and soft tissue infections for 15.6%. Nursing home-acquired bacteremia (NHAB) represented a very small proportion of infections —2.6%— with only two cases identified among the 3,140 residents surveyed. The prevalence of bloodstream infections was even lower when considering all participating European facilities: only nine out of 1,968 healthcare-associated infections (0.5%) were bloodstream infections, among 61,045 residents surveyed [5]. Studies specifically addressing NHAB remain scarce [8, 9]. This condition is rarely identified in epidemiological studies conducted within nursing homes, likely because residents showing general signs of infection are often transferred to acute care settings for further management. Consequently, the most severe infections in nursing home residents, such as NHABs, are largely underreported in prevalence surveys.

In the context of infection prevention and the fight against antimicrobial resistance, the WHO recommends the surveillance of healthcare-associated infections and the implementation of preventive measures [10]. In France, voluntary healthcare institutions participate in an annual three-month surveillance program targeting healthcare-associated bacteremia [11]. As part of this program, participating centers report all cases of healthcare-associated bacteremia, regardless of the acquisition site. Here, we present data on NHAB in France from 2020 to 2024 and describe the characteristics of the affected residents. Our study had two main objectives: first, to assess the magnitude of the issue in nursing homes by identifying the main types of invasive device-associated bacteremia and estimating their incidence rates —particularly those related to intravascular devices and urinary catheters; second, to identify opportunities for improving the prevention of NHAB.

## Methods

Healthcare-associated bacteremia surveillance was conducted annually over a three-month period between January 1st and July 15th in participating healthcare institutions. During the surveillance period, in each participating center, the local infection control practitioner reviewed positive blood cultures to determine whether they met the criteria for healthcare-associated bacteremia, according to a protocol adapted from the HAI-Net ICU ECDC protocol (https://www.ecdc.europa.eu/sites/default/files/documents/HAI-Net-ICU-protocol-v2.2_0.pdf). Briefly, potential contaminants were first excluded. Bacteremia was confirmed when the isolated microorganism was not considered a potential contaminant or, —if it was— when at least two positive cultures were obtained at different times during the same episode, with clinical improvement following targeted antibiotic therapy or catheter removal, if applicable. Next, non-healthcare-associated bacteremias were excluded. These were defined as bacteremias occurring in patients who had not been hospitalized for more than 48 h at the time of blood culture collection, had no recent exposure to healthcare settings (within the past 6 months), or —if such exposure had occurred—, if it was unrelated to the current episode. Based on the patient’s healthcare history, bacteremia was classified as nosocomial (if acquired in a healthcare facility), nursing home-acquired (if acquired in a nursing home), or community/home-acquired (if associated with care received outside institutional settings). Only bacteremias acquired in nursing home (NHAB) were included in the study, and their characteristics were determined using clinical and biological data. For each NHAB case, patient characteristics were collected, including sex, age, presence or absence of immune impairment due to disease or immunosuppressive therapy (e.g., organ transplants, autoimmune disease, or cancer), neutropenia (< 500 PMN/mm^3^), COVID-19 status, presence of actively progressing cancer, presence of hematologic malignancies (e.g., anemia, leukemia, lymphoma, or myelodysplasic syndromes), and death within 7 days of bacteremia diagnosis. The source of bacteremia was also documented and categorized as one of the following: skin (primary cutaneous infection or wound superinfection), surgical site, lungs, urinary tract, intravascular device, intra-abdominal infection, digestive tract, or gastrointestinal translocation. The associated microorganisms were also recorded. For urinary tract-associated NHABs, a recent history of urinary catheterization was investigated. If applicable, details regarding the urinary catheter were collected, including date of insertion, catheter type, and the time between insertion and bacteremia onset. Similarly, for intravascular device-associated NHABs, data on catheter type, insertion date, and time to bacteremia onset were collected.

All data were entered into a secure national web-based tool. Data analysis was conducted by the national team using R software (version 3.6.1 on Ubuntu). NHABs were acquired in nursing homes either located within or outside participating healthcare institutions. For bacteremias acquired in nursing homes within participating institutions, the infection control practitioner had access to the number of resident-days during the surveillance period, allowing the calculation of NHAB incidence rates per 1,000 resident-days. For NHABs acquired in nursing homes outside participating healthcare institutions, resident-day data were not available, preventing incidence rate calculation. The study therefore included (1) all NHAB cases and characteristics of affected residents, regardless of nursing home location; and (2), NHAB incidence rates, but only for nursing homes for which resident-day date were available.

Pearson's chi-squared test and Kruskal–Wallis test were used to compare categorical and numerical variables, respectively. All statistical analyses were two-tailed, with a significant threshold of *p*-value < 0.05. Analyses were performed using Strata version 10.0 software (Stata Corp., College Station, TX, USA).

## Results

A total of 1,233 healthcare institutions participated in the surveillance program at least once during the five survey campaigns, including 243 (19.7%) that had an affiliated nursing home. These institutions were distributed across all the regions from the national territory (Supplementary Fig. 1), and included 72 university and/or regional hospitals, 408 general hospitals, 366 short-stay private clinics, 24 oncology centers, 192 rehabilitation care centers, 23 psychiatric centers, 26 home care centers, 51 long-stay local centers, and 57 chronic dialysis centers. The participating centers represented 46.2% of the French healthcare institutions (based on base SAE 2022). During the surveillance period, a total of 2,117 nursing home-acquired bacteremia (NHAB) cases were documented, originating from all regions of France (Supplementary Fig. 1).

### Resident characteristics

 (Table 1). The median age of residents with NHAB was 87.0 years. The male-to-female ratio was 0.93, with 1,017 males (48.0%) and 1,098 females (52.0%). Impaired immunity was reported in 8.5% (*n* = 167) of cases, while actively progressing cancer was present in 12,4% (*n* = 243). Co-infection with SARS-CoV-2 varied significantly over the study period, with rates of 6.5% in 2020, 8.8% in 2021, 11.7% in 2022, 4.7% in 2023, and 5.5% in 2024 (*p* = 0.002). Mortality within seven days following the onset of bacteremia was reported in 18.2% (*n* = 370) of cases. Residents with bacteremia associated with an intravascular device differed significantly from other residents in terms of age, sex ratio, and co-morbidities: they were younger, more often female, and more frequently presented with impaired immunity and cancer (Table 1; *p* < 0.001). Mortality within seven days before the onset of bacteremia was more frequent among residents with a bronchopulmonary source of infection compared to those with other sources.

**Table 1:** Characteristics of residents with nursing home-acquired bacteremia, by source of infection (2020–2024)

Number of residents with nursing home-acquired bacteremia, by source of infection (%^1^)												
	All	Urinary tract	with urinary catheterization		Pulmonary tract	Digestive tract	Skin and soft tissues	Osteo-articular infection	Intra-vascular device^3^	Endocarditis	Others	Unknown
			no	yes								
N	2,117	1,104 (52.1)	570	386	252 (11.9)	210 (9.9)	173 (8.2)	37 (1.7)	38 (1.8)	23 (1.1)	29 (1.4)	251 (12.4)
Age (y)^4^	87	87	87	85	89	88	87	86	77.5	87	88	87
> 85 y (%)	1,238 (58.5)	609 (55.2)	339 (59.5)	187 (48.4)	177 (70.2)	138 (65.7)	100 (57.8)	21 (56.8)	13 (34.2)	13 (56.5)	19 (65.5)	148 (59.0)
Sex ratio ^5^	0.93	1.05	0.58	3.54	0.84	0.63	0.62	1.47	0.58	1.56	0.81	1.02
Males (%)	1017 (48.8)	565 (51.2)	209 (36.7)	301 (78.0)	115 (45.6)	81 (38.6)	66 (38.2)	22 (59.5)	14 (36.8)	14 (60.9)	13 (44.8)	127 (50.6)
Impaired immunity	167 (8.5)	66 (6.4)	28 (5.2)	28 (7.8)	22 (9.3)	15 (7.6)	18 (11.3)	3 (9.1)	21 (60.0)	1 (4.3)	1 (3.8)	20 (9.1)
Cancer	243 (12.4)	116 (11.3)	46 (8.6)	58 (15.9)	17 (7.2)	34 (17.4)	11 (6.7)	5 (15.6)	24 (64.9)	2 (9.1)	1 (3.7)	33 (14.9)
Co-infection with SARS-CoV2												
2020	19 (6.5)	8 (4.7)	2 (2.2)	5 (11.4)		1 (5.3)		1 (20.0)	1 (25.0)			8 (30.8)
2021	36 (8.8)	15 (6.7)	6 (5.4)	6 (7.1)	7 (21.9)	2 (5.6)	5 (12.5)		2 (15.4)	2 (40.0)	1 (14.3)	2 (4.1)
2022	45 (11.7)	17 (9.0)	7 (6.9)	8 (11.3)	3 (8.1)	4 (8.3)	7 (26.9)	3 (23.1)	0	1 (14.3)		10 (16.7)
2023	16 (4.7)	5 (2.8)	1 (1.1)	3 (4.2)	3 (5.3)	3 (8.3)	1 (4.3)		3 (50.0)			1 (2.9)
2024	16 (5.5)	8 (5.3)	5 (6.2)	2 (4.1)	4 (9.8)	2 (8.7)	1 (3.6)				1 (33.3)	
Death^6^	370 (18.2)	114 (10.9)	51 (9.4)	43 (11.6)	80 (32.5)	45 (22.2)	34 (20.4)	5 (13.9)	8 (21.6)	4 (17.4)	3 (10.7)	77 (31.8)

^1^Percentages were calculated excluding missing data, ^2^ Residents with urinary catheterization within seven days prior to the onset of bacteremia, ^3^Intravascular device-associated bacteremia, ^4^ Median value, ^5^ Number of males/number of females, ^6^ Death within seven days after the onset of bacteremia.

The residents did not differ in terms of age, sex ratio, prevalence of impaired immunity or actively progressing cancer, or in their seven-day outcome following the onset of bacteremia, according to the region in which they were located (Supplementary Table 1). Among all resident characteristics, only the prevalence of COVID-19 co-infection among varied significantly, with the highest rate observed in the Grand Est region (*p* = 0.002). These regional differences should be interpreted with caution, as they may reflect substantial variation in COVID-19 testing practices across healthcare institutions.

#### Bacteremia source

Among the 2,117 NHAB cases, the four primary sources were urinary tract (*n* = 1,104; 52.1%), pulmonary tract (*n* = 252; 11.9%), digestive or abdominal infections including gastrointestinal translocations (*n* = 210; 9.9%), and primary cutaneous infections or superinfections of skin wound (*n* = 173; 8.2%) (Table 2). The origin of bacteremia was undetermined in 251 cases (11.9%). The distribution of bacteremia sources was relatively consistent across regions, with no major differences observed (Supplementary Table 2; *p* = 0.015). For 211 of the 251 residents in whom no entry site could be identified, the presence or absence of an intravascular catheter within seven days prior to bacteremia onset was documented. Among these, 58 residents had carried an intravascular device within that period (including 12 with a central veinous catheter and 46 with a short veinous peripheral catheter). The distribution of NHAB sources remained stable throughout the study period. A total of 608 NHAB cases (28.7%) were likely associated with an invasive device, including 38 linked to intravascular devices and 570 urinary tract-associated cases in residents who had undergone urinary catheterization within seven days prior to bacteremia onset (Table 1). None of the NHAB cases with a pulmonary source was associated with a recent history of invasive or non-invasive ventilation.

**Table 2:** Sources of the 2,117 nursing home-acquired bacteremia (NHAB) cases (2020–2024)

Number of the NHAB (/100 NHABs) by survey period						
Sources of the NHAB	2020–2024	2020	2021	2022	2023	2024
Urinary (/100 NHABs)	1,104 (52.1)	286 (54.4)	228 (53.8)	209 (50.1)	203 (51.4)	178 (50.1)
Broncho-pulmonary (/100 NHABs)	252 (11.9)	65 (12.4)	32 (7.5)	40 (9.6)	63 (15.9)	52 (14.6)
Digestive (/100 NHABs)	210 (9.9)	48 (9.1)	37 (8.7)	50 (12.0)	44 (11.1)	31 (8.7)
Skin and soft tissues (/100 NHABs)	173 (8.2)	44 (8.4)	40 (9.4)	28 (6.7)	28 (7.1)	33 (9.3)
Intravascular devices (/100 NHABs)	38 (1.8)	7 (1.3)	15 (3.5)	1 (0.2)	6 (1.5)	9 (2.5)
Peripherally inserted central catheter	11 (0.5)	1 (0.2)	2 (0.5)	1 (0.2)	1 (0.3)	6 (1.7)
Implantable venous access device	13 (0.6)	2 (0.4)	6 (1.4)		3 (0.8)	2 (0.6)
Short-term central veinous catheter	3 (0.1)	2 (0.4)	1 (0.2)			
Arterial catheter	1 (< 0.1)		1 (0.2)			
Midline	3 (0.1)		1 (0.2)		1 (0.3)	1 (0.3)
Short venous peripheral catheter	5 (0.2)	2 (0.4)	2 (0.5)		1 (0.3)	
Dialysis catheter	2 (0.1)		2 (0.5)			
Dialysis fistula (/100 NHABs)	3 (0.1)	1 (0.2)	1 (0.2)			1 (0.3)
Endocarditis (/100 NHABs)	23 (1.1)	5 (1.0)	5 (1.2)	8 (1.9)	2 (0.5)	3 (0.8)
Osteo-articular infection (/100 NHABs)	37 (1.7)	8 (1.5)	6 (1.4)	13 (3.1)	5 (1.3)	5 (1.4)
Surgical site (/100 NHABs)	4 (0.2)	1 (0.2)		2 (0.5)		1 (0.3)
Others	22 (1.0)	8 (1.5)	6 (1.4)	3 (0.7)	4 (1.0)	1 (0.3)
Unknown (/100 NHABs)	251 (11.9)	53 (10.1)	54 (12.7)	63 (15.1)	40 (10.1)	41 (11.5)

#### Microorganisms responsible for bacteremia

**Fig. 1:**
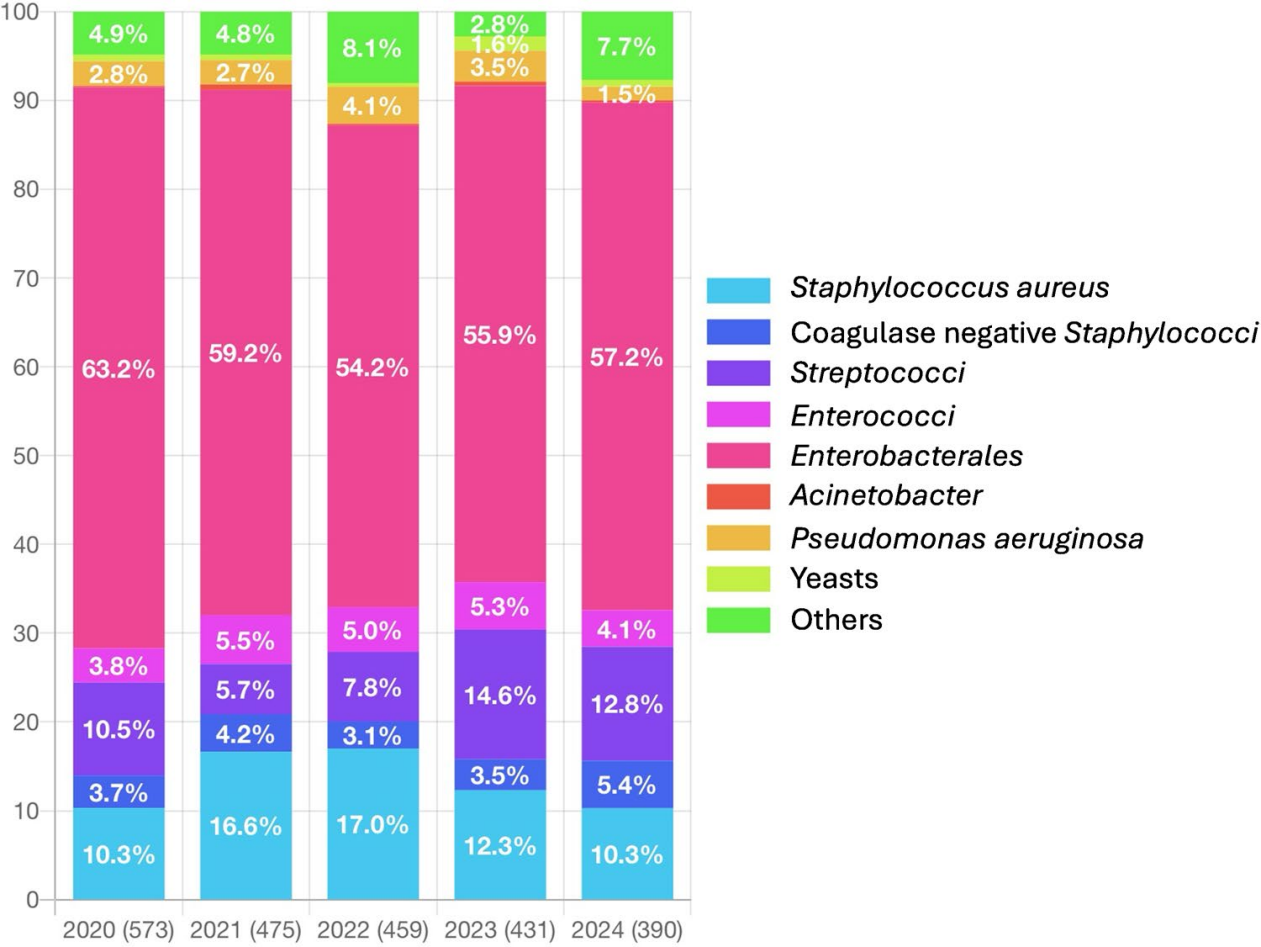
Distribution of the microorganisms associated with the 2,117 nursing home-acquired bacteraemia cases (2020–2024)

Over the entire study period, the most frequently isolated pathogens were *Enterobacterales* (*n* = 1,356; 64.0%), *Staphylococcus aureus* (*n* = 309; 14.6%) and *Streptococci* (*n* = 224; 10.6%). The distribution of microorganisms varied significantly according to the source of bacteremia: *Enterobacterales* predominated in urinary and digestive tract-associated bacteremia; *S. aureus* was most frequently associated with intravascular device-related infections, osteoarticular infections, skin and soft tissue infections, and endocarditis; and *S. pneumoniae* was the leading pathogen in bronchopulmonary-associated bacteremia (*p* < 0.001). The distribution of microorganisms involved in NHAB changed significantly over the study period, with a marked increase in the proportion of *S. aureus*-associated NHAB during the 2021 and 2022 surveillance periods, accompanied by a concurrent decrease in *Streptococci*-associated NHAB (Table 3 and Fig. 1; *p* < 0.001). Among the 309 *S. aureus* isolates, 97 (32.2%) were methicillin-resistant (MRSA) (8 data points missing). Among the 1,356 *Enterobacterales* isolates, 214 (16.0%) exhibited reduced susceptibility or resistance to third-generation cephalosporins (21 data points missing), including three carbapenemase-producing strains (one *bla*_*KPC*_ and two *bla*_*NDM*_). Of the 69 identified *P. aeruginosa* isolates, 11 (17.2%) showed reduced susceptibility or resistance to carbapenems (5 data points missing). Overall, 322 of the 2,117 NHAB cases (15.2%) were associated with multidrug-resistant organisms (MDROs), with no significant trend observed over the study period. However, the proportion of MDRO-associated bacteremia varied significantly according to the source of infection (*p* < 0.001), ranging from 6.7% for bronchopulmonary-associated bacteremia to 19.3% for urinary tract-associated cases. The microorganisms associated with NHAB also differed by region, with significant variation in the proportion of *Enterobacterales* exhibiting reduced susceptibility or resistance to third-generation cephalosporins, as well as in the overall proportion of MDRO-associated bacteremia. In both cases, the lowest rates were observed in the Normandy region and the highest in the Île-de-France region (Supplementary Table 3; *p* < 0.001).

**Table 3:** Microorganisms associated with nursing home-acquired bacteraemia by source (2020–2024)

Number of bacteraemia (B) by source (%^1^)												
	All	Urinary tract	with urinary catheterization^2^		Pulmonary tract	Digestive tract	Skin and soft tissues	Osteo-articular infection	Intra-vascular device^3^	Endocarditis	Others	Unknown
			no	yes								
N bacteraemia	2,117	1,104	570	386	252	210	173	37	38	23	29	251
Staphylococci (/100 B)	400 (18.9)	97 (8.8)	25 (4.4)	62 (16.1)	54 (21.4)	6 (2.9)	76 (43.9)	24 (64.9)	23 (60.5)	9 (39.1)	19 (65.5)	92 (36.6)
*S. aureus* (/100 B)	309 (14.6)	84 (7.2)	18 (3.1)	58 (15.0)	35 (13.9)	6 (2.9)	60 (34.7)	21 (56.8)	10 (26.3)	7 (30.4)	15 (51.7)	71 (28.3)
MRSA (/100 *S. aureus*)	97 (32.2)	41 (51.9)	5 (29.4)	32 (59.3)	7 (20.0)	2 (50.0)	17 (28.3)	7 (35.0)	3 (30.0)	1 (14.3)	2 (13.3)	17 (23.9)
CoNS^3^	91 (4.3)	13 (1.2)	7 (1.2)	4 (1.0)	19 (7.5)		16 (9.2)	3 (8.1)	13 (34.2)	2 (8.7)	4 (13.8)	21 (8.4)
Streptococci (/100 B)	224 (10.6)	23 (2.1)	10 (1.7)	11 (2.8)	84 (33.3)	8 (3.8)	59 (34.1)	6 (16.2)	1 (2.6)	9 (39.1)	2 (6.9)	44 (17.5)
*S. pneumoniae* (/100 B)	47 (2.2)				44 (17.5)			1 (2.7)				2 (0.8)
*S. agalactiae* (/100 B)	34 (1.6)	10 (0.9)	5 (0.9)	4 (1.0)	6 (2.4)		8 (4.6)	1 (2.7)				9 (3.6)
*S. pyogenes* (/100 B)	35 (1.6)	0			6 (2.4)		25 (14.4)		1 (2.6)			3 (1.2)
Other (/100 B)^4^	120 (5.7)	13 (1.2)	5 (0.9)	7 (1.8)	28 (11.1)	8 (3.8)	26 (15.0)	4 (10.8)		9 (39.1)	2 (6.9)	30 (11.9)
Enterococci (/100 B)	110 (5.2)	58 (5.2)	17 (3.0)	36 (9.3)	5 (2.0)	25 (11.9)	3 (1.7)	1 (2.7)	2 (5.3)	4 (17.4)	1 (3.4)	11 (4.4)
*Enterobacterales* (/100 B)	1,356 (64.0)	957 (86.7)	512 (89.8)	308 (79.8)	82 (32.5)	171 (81.4)	27 (15.6)	6 (16.2)	8 (21.0)	1 (4.3)	6 (20.7)	98 (39.0)
ERC3G^5^ (/100 *E*^6^)	214 (16.0)	167 (17.7)	70 (13.8)	68 (22.6)	9 (11.3)	23 (13.8)	3 (11.1)		1 (12.5)		2 (33.3)	9 (9.2)
EPC (/100 *E*)	3 (0.2)	1 (0.1)		1 (0.3)		2 (0.9)						0
*E. coli* (/100 B)	901 (42.6)	651 (59.0)	388 (68.1)	165 (42.7)	54 (21.4)	116 (55.2)	6 (3.5)	2 (5.4)	4 (10.5)		2 (6.9)	66 (26.3)
*Klebsiella* (/100 B)	161 (7.6)	110 (10.0)	43 (7.5)	47 (12.2)	9 (3.6)	24 (11.4)	4 (2.3)	1 (2.7)	2 (5.3)		2 (6.9)	9 (3.6)
*Enterobacter* (/100 B)	45 (1.9)	27 (2.4)	6 (1.0)	20 (5.2)	2 (0.8)	8 (3.8)		2 (5.4)	2 (5.3)			4 (1.6)
*Proteus* (/100 B)	171 (8.1)	123 (11.1)	64 (11.2)	47 (12.2)	12 (4.8)	8 (3.8)	12 (6.9)	1 (2.7)		1 (4.3)	2 (6.9)	12 (4.8)
Other (/100 B)	78 (3.7)	46 (4.2)	11 (1.9)	29 (7.5)	5 (2.0)	15 (7.1)	5 (2.9)					7 (2.8)
*Acinetobacter* (/100 B)	8 (0.3)	4 (0.4)	3 (0.5)	1 (0.2)		1 (0.5)			2 (5.3)			1 (0.4)
*P. aeruginosa* (/100 B)	69 (3.2)	34 (3.1)	9 (1.6)	22 (5.7)	13 (5.2)	5 (2.4)	6 (3.5)	1 (2.7)	2 (5.3)	1 (4.3)	1 (3.4)	6 (2.4)
PARC (/100 *P*)^7^	11 (17.2)	5 (16.1)		4 (20.0)	1 (8.3)		3 (50.0)		1 (50.0)	1 (100)		
Yeasts (/100 B)	19 (0.9)	8 (0.7)	3 (0.5)	5 (1.3)	1 (0.4)	3 (1.4)	1 (0.6)		3 (7.9)			3 (1.2)
Others (/100 B)	128 (6.0)	35 (3.2)	19 (3.3)	13 (3.4)	23 (9.1)	25 (11.9)	20 (11.6)	1 (2.7)	2 (5.3)		2 (6.9)	18 (7.7)
MDROs (/100 B)^8^	322 (15.2)	213 (19.3)	75 (13.2)	104 (26.9)	17 (6.7)	25 (11.9)	23 (13.3)	7 (18.9)	5 (13.2)	2 (8.9)	4 (13.8)	26 (10.3)

^1^Percentages are calculated excluding missing data, ^2^Bacteremia associated with urinary catheterization within seven days prior to onset of bacteremia, ^3^Coagulase negative *Staphylococci*, ^4^Other *Streptococci*, ^5^*Enterobacterales*, ^6^*Enterobacterales* with reduced susceptibility or resistance to third-generation cephalosporins (per 100 ^5^*Enterobacterales* isolates), ^7^*P. aeruginosa* with reduced susceptibility or resistance to carbapenems (per 100 *P. aeruginosa* isolates), ^8^Multiresistant microorganisms as the sum of MRSA, ERC3G and PARC per 100 NHABs

#### Bacteremias associated with urinary catheterization

Among the 1,104 residents who developed urinary tract-associated bacteremia, information on recent urinary catheterization history was available for 856 cases (77.5%). Of these, 386 residents (45.1%) had a recent history of urinary catheterization. The median age of these residents was 85.0 years (Table 1). The male-to-female ratio was 3.54, with 301 males and 78 females, a significantly different distribution compared to other residents (78.7% of males with urinary catheter-associated bacteremia vs 36.7% males among residents with urinary-source bacteremia without recent catheterization or another source; *p* < 0.001). Among these residents, 7.8% (*n* = 28) had impaired immunity, 15.9% (*n* = 58) had actively progressing cancer, and 11.6% (*n* = 43) died within seven days of bacteremia onset. Urinary catheter type was documented in 301 cases (78.0%), with the following distribution: long-term indwelling urinary catheter in 274 cases (91.0%), intermittent catheterization in 17 cases (5.6%), and suprapubic catheter in ten cases (3.3%). The microorganism involved in urinary catheter-associated bacteremia were primarily *Enterobacterales* (*n* = 308; 79.8%) and *S. aureus* (*n* = 58; 15.0%) (Table 3). They differed significantly from those responsible for urinary-source bacteremia without recent catheterization, showing a higher proportion of *S. aureus* (15.0% vs 3.1%), *Enterococci* (9.3% vs 3.0%), *P. aeruginosa* (5.7% vs 1.6%), *Klebsiella* (12.2% vs 7.5%) and *Enterobacter* (5.2% vs 1.0%), and a lower representation of *E. coli* (42.7% vs 68.1%) (*p* < 0.001). Among the 386 catheter-associated bacteremias, 104 (26.9%) were caused by MDROs, including 32 MRSA, 68 *Enterobacterales* with reduced susceptibility or resistance to third-generation cephalosporins, and four *P. aeruginosa* with reduced susceptibility or resistance to carbapenems. The prevalence of MDROs in urinary catheter-associated bacteremia was significantly higher than in urinary-source bacteremia without recent catheterization (26.9% vs 13.2%; *p* < 0.001).

#### Bacteremias associated with intravascular device

Residents who developed bacteremia associated with an intravascular catheter differed from those with bacteremia of other sources. They had a lower median age (77.5 years), a significantly higher prevalence of impaired immunity (66.7% vs 7.4%; *p* < 0.001), and a markedly higher prevalence of actively progressing cancer (60.0% vs 11.1%; *p* < 0.001). Death within seven days following bacteremia onset was reported in eight cases (21.6%). The intravascular devices most frequently involved in NHAB were implanted vascular access devices (IVAD; *n* = 13; 34.2%) and peripherally inserted central catheters (PICC; *n* = 11; 28.9%) (Table 2). The microorganisms responsible for intravascular device-associated-bacteremia were predominantly *Staphylococci* (*n* = 23; 60.5%) (Table 3). *S. aureus* was involved in ten cases (26.3% of catheter related NHAB), with 30.0% of isolates being MRSA.

#### Incidence data

Out of the 1,233 healthcare institutions participating in the national survey program, 243 had a nursing home and monitored healthcare-associated bacteremia within it. This enabled estimation of the incidence of NHAB. The number of participating nursing homes per year ranged from 81 to 141 (Table 4). The overall incidence rate of the bacteremia (all sources combined) acquired in nursing homes over the five-year period was 0.009 per 1,000 resident-days, varying from 0.004 to 0.012 depending on the year. This rate was 15 times lower than that observed in long-stay units and 82 times lower than in medical wards. The only invasive device-associated bacteremias were those related to urinary catheterization. The incidence rate of these cases was 0.005 per 1,000 resident-days, ranging from 0.002 to 0.008 per year. Again, this rate was 15 times lower than in long-stay units and 35 times lower than in medical wards. Over the 5-year period, the incidence rate of bacteremia in participating nursing homes remained stable, contrasting with fluctuations seen in intensive care and short-stay units, which were impacted by the COVID-19 pandemic.

**Table 4:** Evolution of incidence rates (IR) of bacteraemia acquired in the 1,233 participating centers (2020–2024)

	Campaigns	Intensive care units	Haematological units	Oncological units	Medical units	Surgical units	Rehabilitation units	Long-stay units	Nursing homes
N participating centers	2020	167	52	160	436	367	410	166	141
	2021	168	47	135	405	343	364	137	97
	2022	174	45	134	385	312	354	127	88
	2023	166	50	136	369	287	329	123	84
	2024	164	48	129	355	289	305	120	81
N patient-days or resident-days	2020	225,075	93,147	312,809	3185,309	1436,420	2094,631	831,710	2257,334
	2021	299,543	97,295	283,253	3051,537	1462,719	1700,599	676,264	1462,921
	2022	237,178	88,143	268,478	3121,176	1345,302	1783,815	620,728	1402,846
	2023	232,640	90,574	262,309	3094,277	1290,727	1694,316	622,175	1246,225
	2024	216,404	84,772	253,831	2951,407	1221,182	1587,461	586,179	1329,233
	5 years	1210,840	453,931	1380,680	15,403,706	6756,350	8860,822	3337,056	7691,935
Incidence rateR of bacteremias (all sources) per 1000 PDs/RDs	2020	3.981	3.886	1.992	0.668	0.666	0.261	0.124	0.012
	2021	4.941	4.584	1.610	0.746	0.632	0.285	0.143	0.008
	2022	4.473	5.321	2.183	0.720	0.665	0.293	0.111	0.007
	2023	3.443	4.470	2.013	0.748	0.776	0.307	0.145	0.012
	2024	3.701	5.485	2.151	0.830	0.803	0.334	0.157	0.004
	5 years	4.162	4.769	1.984	0.741	0.704	0.294	0.135	0.009
P*	< 0.001	0.042	0.353	0.265	0.081	0.006	0.142	0.344	0.738
Incidence rate of bacteremias associated with a central line per 1000 PDs/RDs	2020	1.075	1.449	0.972	0.116	0.104	0.054	0.004	0
	2021	1.108	1.665	0.763	0.121	0.111	0.053	0.012	0
	2022	0.995	1.611	0.931	0.116	0.120	0.047	0.003	0
	2023	0.726	1.502	0.904	0.127	0.152	0.052	0.006	0
	2024	0.801	1.427	1.028	0.123	0.145	0.061	0.007	0
	5 years	0.952	1.533	0.918	0.120	0.125	0.053	0.006	0
P*	0.006	0.280	0.248	0.255	0.733	0.205	0.856	0.766	NC
Incidence rate of bacteremias associated with a peripheral veinous catheter line per 1000 PDs/RDs	2020	0.116	0.097	0.064	0.076	0.038	0.010	0.002	0
	2021	0.124	0.123	0.042	0.089	0.039	0.009	0.004	0
	2022	0.097	0.068	0.060	0.094	0.046	0.010	0.002	0
	2023	0.155	0.088	0.042	0.098	0.054	0.012	0.002	0
	2024	0.092	0.189	0.051	0.120	0.057	0.009	0.002	0
	5 years	0.117	0.112	0.052	0.095	0.046	0.010	0.002	0
P*	0.328	0.658	0.248	0.867	0.012	0.092	0.915	0.804	NC
Incidence rate of bacteremias associated with a urinary catheterization per 1000 PDs/RDs	2020	0.227	0.322	0.221	0.151	0.200	0.106	0.064	0.008
	2021	0.290	0.329	0.247	0.172	0.151	0.131	0.075	0.004
	2022	0.266	0.363	0.291	0.168	0.190	0.123	0.060	0.004
	2023	0.228	0.331	0.335	0.174	0.192	0.133	0.074	0.006
	2024	0.282	0.413	0.339	0.203	0.193	0.144	0.090	0.002
	5 years	0.260	0.350	0.283	0.173	0.185	0.126	0.072	0.005
P*	0.545	0.696	0.002	0.027	0.004	0.229	0.180	0.974	0.774
Incidence rate of bacteremias (all sources) involving *S. aureus* per 1000 PDs/RDs	2020	0.484	0.290	0.195	0.138	0.086	0.030	0.013	0
	2021	0.588	0.247	0.247	0.178	0.085	0.046	0.013	0.001
	2022	0.527	0.272	0.313	0.157	0.092	0.044	0.008	0
	2023	0.370	0.254	0.252	0.165	0.112	0.031	0.019	0.002
	2024	0.287	0.330	0.256	0.165	0.117	0.046	0.024	0.001
	5 years	0.461	0.278	0.251	0.160	0.097	0.039	0.015	0.001
P*	< 0.001	0.695	0.353	0.243	0.239	0.023	0.039	0.248	0.630

NC not calculable, *Kruskal–Wallis test.

#### Nursing home-acquired bacteremia and nursing home-type

The 243 institutions that had a nursing home and monitored healthcare-associated bacteremia within it consisted of 12 university/regional hospitals, 181 general hospitals, 25 local hospitals, 11 rehabilitation centers, four psychiatric centers, three private clinics, and 14 nursing homes. The nursing homes in these participating centers varied in size, with the highest number of beds in nursing homes within university/regional hospitals (*n* = 240) and general hospitals (200 beds), and the lowest in independent nursing homes (73 beds) and clinics (64 beds). Resident characteristics were similar across nursing home types. However, when comparing NHAB cases from residents from nursing homes located in university/regional hospitals (*n* = 8), general hospitals (*n* = 52), and independent nursing homes (*n* = 8), catheter-related bacteremia was significantly overrepresented in independent nursing homes (3/8 vs 0/60; *p* < 0.001).

## Discussion

Our study, based on data from 1,233 healthcare institutions, provides detailed descriptive insights into bacteremia acquired in nursing homes (NHAB). Specifically, we present robust incidence data from a three-month survey conducted in 243 nursing homes. To the best of our knowledge, these data offer recent and valuable insights into bacteremia in the unique nursing home setting, where such data remain scarce [6, 7, 9].

The characteristics of residents with bacteremia differ from those reported for the general nursing home population. According to data from the recent European point prevalence survey HALT-4 [6, 7], the residents with bacteremia in our study were younger and more frequently male. Common risk factors for healthcare-associated infections were commonly observed, particularly a high prevalence of residents with actively progressing cancer and/or impaired immunity.

One-day prevalence studies conducted in nursing home settings typically report urinary tract infections, pneumonia, and skin infections as the most common infections [3–6]. In our study, the main sources of NHAB reflect these distributions. We observed a notable proportion of NHAB cases for which the portal of entry was not identified by clinicians. It is possible—although not demonstrated by our data—that in some nursing homes, limited access to comprehensive diagnostic facilities may have reduced clinicians’ ability to precisely determine the mechanisms leading to bacteremia. A survey among clinicians could help clarify this issue.

Our analysis of 2,117 NHAB cases highlights a substantial proportion of invasive device-associated bacteremia (approximately one in five cases), predominantly urinary catheter-associated bacteremia, with only a minimal contribution from intravascular catheter-associated bacteremia. Due to the design of our study (which was not patient-based and did not involve monitoring the characteristics of all residents—only those with NHAB were thoroughly documented), we were unable to determine residents’ exposure to urinary or vascular catheterization. The 2024 national point prevalence survey conducted in 102,166 French residents across 1,288 nursing homes, reported that 1.75% of residents had a long-term indwelling urinary catheter on the day of the survey, and 0.22% had an intravascular catheter [7]. The greater exposure of residents to urinary catheterization compared to intravascular catheterization (eight times higher) should explain, at least in part, the predominance of urinary catheter-associated bacteremia among bacteremias related to invasive devices. The overrepresentation of urinary catheter-associated bacteremia confirms catheterization as a notable infection risk factor in residents. These NHABs were predominantly observed in male residents with long-term catheters. Of particular concern, our data revealed that one in four urinary catheter-associated bacteremias were caused by a MDRO. These findings underscore the growing challenge of managing infections caused by MRSA, *Enterobacterales* with reduced susceptibility or resistance to third-generation cephalosporins, and *P. aeruginosa* with reduced susceptibility or resistance to carbapenems. We recommend that local infection control teams closely monitor catheterization practices to identify any deviations from current infection prevention guidelines—particularly regarding the quality of antisepsis prior to catheter insertion and strict adherence to aseptic technique. In addition, given the high bacteria load in the urine of infected residents and the potential for environmental contamination during care, our results should serve as a warning to healthcare workers about the risk of MDRO transmission between residents.

Intravascular device-associated bacteremia accounted for only 2% of NHAB cases—a proportion markedly lower than that observed in bacteremic patients hospitalized in short-stay units, where such infections represent up to 30% of healthcare-associated bacteremias [11]. Resident exposure to intravascular devices is low (0.22% according to the 2024 national point prevalence survey conducted in France [7]), and in half of the cases, the catheters involved are IVAPs. This limited exposure to intravascular catheters explains the very low proportion of intravascular device-associated bacteremias acquired in nursing homes. In our study, the devices were primarily PICCs and IVADs, used mostly in residents with cancer. The time between catheter insertion and the onset of clinical signs of infection exceeded seven days in all cases. This raises concerns about adherence to aseptic techniques during line manipulations and catheter dressing changes, which are performed long after catheter insertion. If aseptic precautions are not strictly followed during handling procedures—such as connections, disconnections, blood draws, or flushing—microorganisms from the hands of healthcare workers may enter the lines. These microorganisms can then migrate endoluminally to the catheter and cause late-onset bacteremias [12, 13]. In our study, *Staphylococci—* key components of skin flora—were the most frequently identified pathogens in intravascular device-associated bacteremias. These findings support the hypothesis that catheter contamination may originate, at least in part, from healthcare workers’ skin flora during catheter handling. Given that such manipulations are likely infrequent in nursing homes, catheter care practices may be suboptimal. We recommend that local infection control teams conduct direct observations of line-handling procedures to identify deviations from current guidelines for preventing of intravascular device-associated bacteremia.

The microorganisms involved in NHAB varied over the course of the study, with an increase in the proportion of bacteremias involving *S. aureus* in 2021 and 2022, while those involving *Streptococci* showed an opposite trend. Our study does not allow us to determine the mechanisms underlying these changes. However, as studies have reported a decrease in respiratory infections during the COVID-19 pandemic, linked to widespread use of surgical masks [14], it seems plausible that bacteremias involving *Streptococci*, particularly *S. pneumoniae*, were affected by the use of surgical masks by healthcare workers and residents during the peak of the pandemic. The increased incidence of pneumonia and bacteremia involving *S. aureus* during the COVID-19 pandemic has been documented in numerous studies [15, 16]. One hypothesis to explain this trend is the immune suppression observed in patients infected with SARS-CoV-2 [17], which may have facilitated the development of bronchopulmonary infections in asymptomatic carriers of *S. aureus*. In our study, co-infection with SARS-CoV-2 was observed in 8.8% of bacteriemic residents in 2021, and 11.7% in 2022. It seems plausible that these high co-infection rates contributed to the increased proportion of *S. aureus* infection during these two survey periods.

Because residents are often transferred to acute care facilities at the onset of clinical signs suggestive of bacteremia, NHABs are generally underreported in studies assessing infection prevalence in this setting. In our study, we benefited from the participation of healthcare institutions involved in the national surveillance program, which extended their monitoring to the nursing homes within their structures. Using data from 243 nursing homes, we were thus able to estimate the current extend of bacteremia acquisition in residents. The incidence rate of NHAB, regardless of source, was low—0.009 per 1,000 residents-days—15 times lower than in long-stay units and 90 times lower than in short-stay medical units. These findings are consistent with Mylotte’s review of NHAB [9]. The incidence rate of bacteremia associated with urinary catheterization over the five-year period was 0.002 per 1,000 resident-days, representing one in five NHAB cases. The incidence rate of bacteremia associated with intravascular devices was zero, confirming that such infections remain rare events among nursing home residents.

Our study has several limitations. First, regarding the calculation of incidence rates, the study design did not allow for an analysis of NHAB incidence rates according to nursing home type or geographic region; a larger surveillance sample involving more nursing homes would have been necessary to achieve this. Second, the assessment of infection risk associated with intravascular catheterization using catheter-days was not performed, and variations in NHAB incidence rates related to catheter use could not be analyzed in relation to potential differences in catheter utilization between nursing homes. We are currently working to improve these aspects with the coordinator of the national PRIMO network, which is dedicated to infection prevention in French nursing homes.

## Conclusion

Given the severity of these infections, surveillance of invasive device-associated bacteremia should be encouraged in nursing homes. Infection surveillance methods using denominators that account for resident exposure to invasive devices should be implemented to improve result analysis. Additionally, our findings highlight key areas for enhancing the prevention of invasive device-associated bacteremia in residents. Regarding the use of invasive devices, healthcare workers’ practices should be examined, and educational tools tailored to their specific needs should be developed.

## Supplementary information

Below is the link to the electronic supplementary material.

ESM 1 (DOCX 428 KB)

## Data Availability

No datasets were generated or analysed during the current study.
