# Supplementary material for: Incidence trends and epidemiology of invasive device-associated bacteremia in French nursing home residents, 2020–2024: Insights from the SPIADI Prospective Multicenter Study
Source: Eur J Clin Microbiol Infect Dis. 2025 Jun 23;44(9):2219–30. doi: 10.1007/s10096-025-05188-4 (PMC12457500; doi:10.1007/s10096-025-05188-4)
Supplement: Supplementary file 1 — (DOCX 428 KB) [file 10096_2025_5188_MOESM1_ESM.docx]

**Supplementary figure 1.** Distribution of the 1,233 participating centers across the country (number of documented nursing home-acquired bacteremia).


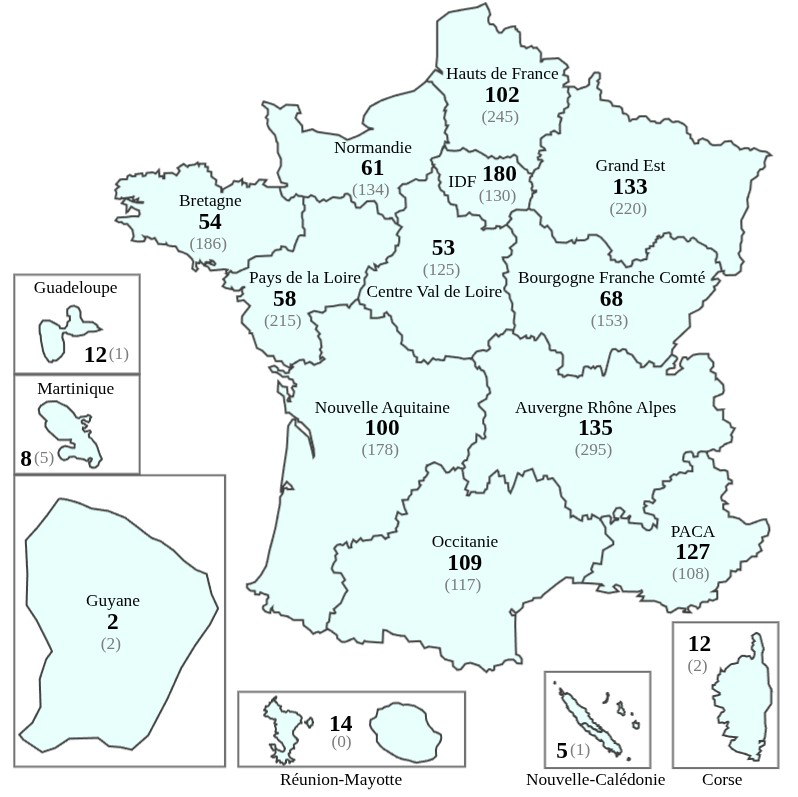


**Supplementary table 1**.

Characteristics of residents with nursing home-acquired bacteraemia by region^1^ (2020-2024).

|  | Number of residents by region (%^2^) | | | | | | | | | | | | |  |
| --- | --- | --- | --- | --- | --- | --- | --- | --- | --- | --- | --- | --- | --- | --- |
|  | Auvergne Rhône  Alpes | Bourgogne Franche Comté | Bretagne | Centre Val de Loire | Grand Est | Hauts de France | Île de France | Normandie | Nouvelle Aquitaine | Occitanie | Pays de la Loire | Provence Alpes Côte d’Azur | p | |
| N | 295 | 153 | 186 | 125 | 220 | 245 | 130 | 134 | 178 | 117 | 215 | 111 |  | |
| Age (y) ^3^ | 87 | 88 | 87 | 87 | 87 | 87 | 88 | 89 | 86 | 87 | 88 | 87 |  | |
| >85 y | 167 | 96 | 104 | 72 | 121 | 146 | 82 | 86 | 98 | 69 | 133 | 60 | 0.666 | |
| Sex ratio ^4^ | 0.78 | 0.82 | 1.09 | 1.08 | 0.85 | 0.78 | 1.06 | 0.67 | 1.08 | 1.21 | 1.01 | 1.16 |  | |
| Males | 129 | 69 | 97 | 65 | 101 | 107 | 67 | 54 | 92 | 64 | 108 | 58 | 0,152 | |
| Impaired immunity | 31  (10.8) | 6  (4.1) | 14  (8.1) | 7  (6.1) | 21  (12.2) | 15  (6.6) | 12  (9.5) | 7  (5.8) | 13  (7.5) | 10  (9.3) | 21  (10.4) | 8  (7.8) | 0.282 | |
| Cancer | 35 (12.1) | 22 (15.3) | 29  (16.8) | 12  (10.2) | 17  (10.3) | 25 (11.2) | 15  (11.9) | 12  (10.0) | 20  (11.7) | 17  (14.9) | 25  (12.3) | 12 (11.8) | 0.787 | |
| Co-infection with SARS-CoV2 (2020-2024) (%) | | | | | | | | | | | | | |  |
|  | 14  (5.3) | 8  (5.8) | 12  (7.9) | 4  (4.2) | 25  (15.6) | 10  (4.6) | 11  (10.5) | 12  (10.2) | 4  (2.8) | 8  (9.3) | 16  (10.4) | 8  (10.2) | 0.002 | |
| Death^5^ | 45 (15.7) | 33 (22.3) | 26  (14.7) | 27  (22.3) | 42 (19.6) | 45 (18.6) | 25  (20.2) | 16  (12.6) | 27  (15.8) | 21  (18.6) | 33  (17.0) | 28 (27.2) | 0.161 | |

^1^we present the data related to the regions where more than 5 bacteraemia were documented; ^2^Percentages are calculated without taking into account the missing data; ^3^ median value; ^4^ N males / N females; ^5^ death within seven days before the onset of bacteremia.

**Supplementary table 2**.

Distribution of nursing home-acquired bacteremia cases by origin and region^1^ (2020-2024).

| Origin of the bacteraemia | Number of the bacteraemia (/100 NHABs) by region (%^2^) | | | | | | | | | | | | |  |
| --- | --- | --- | --- | --- | --- | --- | --- | --- | --- | --- | --- | --- | --- | --- |
|  | Auvergne Rhône  Alpes | Bourgogne Franche Comté | Bretagne | Centre Val de Loire | Grand Est | Hauts de France | Île de France | Normandie | Nouvelle Aquitaine | Occitanie | Pays de la Loire | Provence Alpes  Côte d’Azur | p | |
| N | 295 | 153 | 186 | 125 | 220 | 245 | 130 | 134 | 178 | 117 | 215 | 108 |  | |
| Urinary | 140  (47.4) | 73  (47.7) | 105  (56.4) | 64  (51.2) | 99  (45.0) | 131  (53.5) | 81  (62.3) | 71  (53.0) | 98  (55.0) | 64  (54.7) | 111  (51.6) | 63  (58.3) | 0.015 | |
| Broncho-pulmonary | 38  (12.9) | 20  (13.1) | 20  (10.7) | 10  (8.0) | 22  (10.0) | 27  (11.0) | 9  (6.9) | 22  (16.4) | 22  (12.3) | 14  (12.0) | 33  (15.3) | 13  (12.0) |  |  |
| Digestive | 34  (11.5) | 9  (5.9) | 22  (11.8) | 15  (12.0) | 23  (10.4) | 27  (11.0) | 8  (6.1) | 17  (12.7) | 13  (7.3) | 11  (9.4) | 26  (12.1) | 5  (4.6) |  |  |
| Skin and soft tissues | 23  (7.8) | 16  (10.4) | 6  (3.2) | 11  (8.8) | 21  (9.5) | 15  (6.1) | 14  (10.8) | 13  (9.7) | 21  (11.8) | 6  (5.1) | 17  (7.9) | 8  (7.4) |  |  |
| Intravascular devices | 6  (2.0) | 1  (0.6) | 5  (2.7) | 2  (1.6) | 7  (3.2) | 2  (0.8) | 4  (3.1) |  | 1  (0.6) | 3  (2.6) | 2  (0.9) | 2  (1.8) |  |  |
| Endocarditis | 3  (1.0) |  | 2  (1.1) |  | 4  (1.8) | 2  (0.8) | 3  (2.3) | 1  (0.7) | 2  (1.1) | 3  (2.6) | 2  (0.9) | 1  (0.9) |  |  |
| Others | 14  (4.7) | 4  (2.6) | 8  (4.3) | 5  (4.0) | 5  (2.3) | 8  (3.3) | 2  (1.5) | 3  (2.2) | 1  (0.6) | 6  (5.1) | 5  (2.3) | 5  (4.6) |  |  |
| Unknown | 37  (12.5) | 30  (19.6) | 18  (9.7) | 18  (14.4) | 39  (17.7) | 33  (13.5) | 9  (6.9) | 7  (5.2) | 20  (11.2) | 10  (8.5) | 19  (8.8) | 11  (10.2) |  |  |

^1^we present the data related to the regions where more than 5 bacteraemia were documented; ^2^Percentages are calculated without taking into account the missing data.

**Supplementary table 3**. Microorganisms associated with nursing home-acquired bacteraemia by regions^1^ (2020-2024).

|  | Number of the bacteraemia (/100 NHABs) according to region (%^2^) | | | | | | | | | | | | |  |
| --- | --- | --- | --- | --- | --- | --- | --- | --- | --- | --- | --- | --- | --- | --- |
|  | Auvergne Rhône  Alpes | Bourgogne Franche Comté | Bretagne | Centre Val de Loire | Grand Est | Hauts de France | Île de France | Normandie | Nouvelle Aquitaine | Occitanie | Pays de la Loire | Provence Alpes  Côte d’Azur | p | |
| N bacteraemia | 295 | 153 | 186 | 125 | 220 | 245 | 130 | 134 | 178 | 117 | 215 | 108 |  | |
| *S. aureus*  (/100 B) | 52  (17.6) | 26  (17.0) | 32  (17.2) | 19  (15.2) | 35  (15.9) | 27  (11.0) | 20  (15.4) | 16  (11.9) | 27  (15.2) | 9  (7.7) | 29  (13.5) | 14  (13.0) |  | |
| MRSA^3^  (/100 *S. aureus*) | 13  (27.1) | 9  (34.6) | 12  (37.5) | 4  (21.0) | 12  (34.3) | 8  (30.8) | 13  (65.0) | 5  (31.2) | 8  (44.4) | 3  (33.3) | 6  (22.2) | 4  (28.6) | 0.266 | |
| *Enterobacterales* (/100 B) | 184  (62.4) | 89  (58.2) | 121  (65.0) | 88  (70.4) | 130  (59.1) | 173  (70.6) | 75  (57.7) | 90  (67.2) | 111  (62.3) | 57  (48.7) | 148  (68.8) | 72  (66.7) |  | |
| ERC3G^4^  (/100 *E*^5^) | 31  (17.2) | 11  (12.5) | 24  (19.8) | 12  (13.6) | 19  (14.6) | 35  (20.2) | 23  (30.7) | 5  (5.6) | 8  (7.3) | 11  (19.3) | 19  (13.0) | 15  (20.8) | <0.001 | |
| *P. aeruginosa* (/100 B) | 9  (3.0) | 3  (2.0) | 7  (3.8) | 2  (1.6) | 13  (5.9) | 6  (2.4) | 2  (1.5) | 4  (3.0) | 5  (2.8) | 6  (5.1) | 5  (2.3) | 4  (3.7) |  | |
| PARC  (/100 *P*)^5^ | 3  (60.0) | 1  (33.3) |  |  | 3  (23.1) | 1  (20.0) |  |  |  |  | 2  (50.0) | 1  (25.0) |  | |
| MDROs  (/100 B)^6^ | 47  (15.9) | 21  (13.7) | 36  (19.3) | 16  (12.8) | 34  (15.4) | 44  (17.9) | 36  (27.7) | 10  (7.5) | 16  (9.0) | 14  (12.0) | 27  (12.5) | 20  (18.5) | <0.001 | |

^1^we present the data related to the regions where more than 5 bacteraemia were documented; ^2^Percentages are calculated without taking into account the missing data; ^3^methicillin-resistant *S. aureus*; ^4^*Enterobacterales* with reduced susceptibility or resistance to third generation cephalosporins; ^5^*P. aeruginosa* with reduced susceptibility or resistance to carbapenem (for 100 *P. aeruginosa*); ^6^ Multiresistant microorganisms as the sum of MRSA, ERC3G and PARC per 100 NHABs.
